# Radial Extracorporeal Shock Wave Therapy against *Cutibacterium acnes* Implant-Associated Infections: An in Vitro Trial

**DOI:** 10.3390/microorganisms8050743

**Published:** 2020-05-15

**Authors:** Konstantinos Tsikopoulos, Lorenzo Drago, Georgios Koutras, Panagiotis Givissis, Eleni Vagdatli, Prodromos Soukiouroglou, Paraskevi Papaioannidou

**Affiliations:** 1 1st Department of Pharmacology, School of Medicine, Aristotle University of Thessaloniki, 54124 Thessaloniki, Greece; ppap@auth.gr; 2 Laboratory of Clinical Microbiology, Department of Biochemical Sciences for Health, University of Milan, 20133 Milan, Italy; lorenzo.drago@unimi.it; 3 Department of Physical Therapy, Technological Education Institute of Thessaloniki, 57400 Sindos, Greece; kutrasg@otenet.gr; 4 1st Orthopaedic Department of Aristotle University, G. Papanikolaou General Hospital, Exohi, 57010 Thessaloniki, Greece; pgivissis@gmail.com; 5 Laboratory of Microbiology, Hippokration Hospital, 54642 Thessaloniki, Greece; vagdatli@mls.teithe.gr (E.V.); maksouko@yahoo.gr (P.S.)

**Keywords:** extracorporeal shock wave therapy, biofilms, *Cutibacterium acnes*, *Propionibacterium acnes*, post-operative infections, adjuvant treatment, in vitro

## Abstract

Background: Antibiotic management of low-virulent implant-associated infections induced by *Cutibacterium acnes* may be compromised by multi-drug resistance development, side effects, and increased cost. Therefore, we sought to assess the effects of shock wave therapy against the above pathogen using an in vitro model of infection. Methods: We used a total of 120 roughened titanium alloy disks, simulating orthopedic biomaterials, to assess the results of radial extracorporeal shock wave therapy (rESWT) against *C. acnes* (ATCC 11827) biofilms relative to untreated control. In particular, we considered 1.6 to 2.5 Bar with a frequency ranging from 8–11 Hz and 95 to 143 impulses per disk to investigate the antibacterial effect of rESWT against *C. acnes* planktonic (free-floating) and biofilm forms. Results: Planktonic bacteria load diminished by 54% compared to untreated control after a 1.8-bar setting with a frequency of 8 Hz and 95 impulses was applied (median absorbance (MA) for intervention vs. control groups was 0.9245 (IQR= 0.888 to 0.104) vs. 0.7705 (IQR = 0.712 to 0.864), respectively, *p* = 0.001). Likewise, a statistically significant reduction in the amount of biofilm relative to untreated control was documented when the above setting was considered (MA for treatment vs biofilm control groups was 0.244 (IQR= 0.215–0.282) and 0.298 (IQR = 0.247–0.307), respectively, *p* = 0.033). Conclusion: A 50% biofilm eradication was documented following application of low-pressure and low-frequency radial shock waves, so rESWT could be investigated as an adjuvant treatment to antibiotics, but it cannot be recommended as a standalone treatment against device-associated infections induced by *C. ances.*

## 1. Introduction

*Cutibacterium acnes* (formerly known as *Propionibacterium acnes*) is the most frequently isolated microorganism following shoulder arthroplasty, reaching a prevalence of 38.9% [1]. With the financial burden of revision surgery being around 3.6 times greater compared to that of primary reconstruction [2], shoulder periprosthetic joint infections (PJIs) represent a serious complication not only for patients, but also for orthopedic surgeons.

From a pathogenetical viewpoint, once *C. acnes* adheres to a biomaterial and enters the growth and colonization phases, it is less vulnerable to antimicrobials. Thereafter, biofilms consisting of sessile microorganisms enclosed within a polymer matrix develop, [3,4,5] thereby decreasing the susceptibility of *C. acnes* to antibiotics. In particular, antibiotic dosages up to 1000-times higher than the corresponding minimum inhibitory concentrations are required to eradicate bacteria encapsulated within biofilms [6].

Outcomes following either surgical debridement in conjunction with antibiotics or long-term suppressive chemotherapy are not always optimal [7]. On top of that, side effects related to antibiotic administration can be as high as 22% [8]. Thus, adjuvant nonantibiotic approaches, particularly in terms of ultrasound therapies, may be employed to improve eradication rates [9,10].

Based on the results of prior in vitro laboratory studies, extracorporeal shock wave therapy (ESWT) presents a promising track record of properties against biofilms produced by clinically relevant aerobic microorganisms [11,12]. While the exact mechanism of the bactericidal action of shock waves still remains unclear [13], it has been postulated that shock wave antibacterial activity occurs at a cell wall level through an increase in permeabilization of bacterial cells [14]. Another possible explanation would be the detachment of enclosed bacteria from implant surface following shock wave application. On top of that, improved infection outcomes could be achieved in living organisms as an increased local immune response occurs following shock wave therapy due to revascularization and soft tissue regeneration [15,16,17]. Of note, amongst the two available types of shock wave treatment intended for orthopedic conditions [18] (i.e., focused and radial shock waves), radial extracorporeal shock wave therapy (rESWT) is less extensively investigated and has not been previously tested against anaerobic bacteria. Therefore, we used an in vitro experimental study design to answer the following questions: (1) Is radial extracorporeal shock wave therapy (rESWT) effective against *C. acnes’s* planktonic (free-floating) form? (2) Are biofilm *C. acnes* bacteria susceptible to rESWT?

## 2. Materials and Methods

The standard *C. acnes* ATCC 11827 was utilized because it is the most extensively investigated strain in relevant investigations [19,20,21].

### 2.1. Biomaterials

We employed sandblasted titanium alloy (Ti6Al4V) disks (diameter 4mm; height 2mm; S_a_ > 2 μm) (Online Supplemental Figure S1) resembling orthopedic implants. To remove dirt and ensure sterile working conditions, we rinsed those disks mechanically with low-pressure saline irrigation and subsequently sterilized them at 121 °C prior to use.

### 2.2. Susceptibility Assessment of C. Acnes Planktonic Form to rESWT

A suspension of *C. acnes* was prepared with an optical density of 0.5 McFarland (1.5 × 10^8^ Colony Forming Units (CFU)/mL approximately) and successively inoculated to a final concentration of 10^5^ CFU/mL in a 96-well microtiter plate. We incubated *C. acnes* for 72 h at 37 °C in the presence of sterile titanium disks by using anaerobiosis-generating sachets and the respective jar (Oxoid™ AnaeroGen™ 2.5L; ThermoScientific, Waltham, MA, USA) [21]. Prior to rESWT application (Masterpuls^®^ MP 50 device, Storz Medical AG, Tägerwilen, Switzerland), a thin film of ultrasound gel was considered on the upper surface of each 96 well plate to allow for energy conduction during treatment. Furthermore, in an effort to minimize potential energy loss, care was taken to eliminate any air gaps between the R15 transmitter and the disks. Afterwards, the R15 transmitter was pressed firmly onto the plastic surface and the disks were exposed to radial shock waves at 1.6 to 2.5 Bar with a frequency of 8–11 Hz and 95 to 143 impulses per disk using a standard 15mm head (R15 transmitter). Thereafter, vortexing was performed by means of a microtiter shaking plate and 100 μL from the supernatant of each well was transferred to a new 96-well plate. Subsequently, the XTT ([2–bis(2-methoxy-4-nitro-5-sulfophenyl)-2H-tetrazolium-5-carboxanilide]) proliferation reduction assay was conducted to quantify cell viability [22] and absorbance was read at 450nm with the use of a microplate spectophotometer (Epoch^TM^, BioTek, Vermont, USA).

### 2.3. Mature Biofilm Production Confirmation

**Figure 1:**
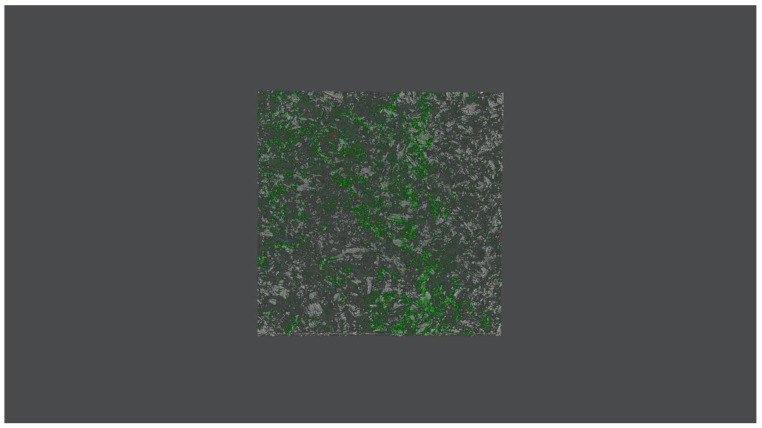
Two-dimensional depiction of a 72-h *C. acnes* biofilm grown on a titanium alloy disk using confocal laser microscopy (Leica model TCS SP5; Leica Microsystems CMS GmbH, Mannheim, Germany). Live bacteria are represented in green and dead cells in red.

Before executing any biofilm experiments, we ensured that *C. acnes* was capable of producing mature biofilms. To achieve this objective, confocal scanning laser microscopy (CSLM) was employed to depict images of organized structures of clustered cells grown on a titanium disk (Figure 1). To illustrate, *C. acnes* biofilms were grown on sandblasted titanium alloy disks and, following careful rinsing with sterile saline, they were subjected to staining for 15 min using Filmtracer™ LIVE/DEAD™ Biofilm Viability Kit (Thermo Fisher Diagnostics SpA). To be more precise, 3 μL of SYTO9 allowing for permeation of living cell membranes and 3 μL of propidium iodide, capable of staining only damaged membranes of dead cells, were added to 1 mL of filter-sterilized water. Then, incubation, washing with sterile saline, and examination with an upright TCS SP8 (Leica Microsystems CMS GmbH, Mannheim, Germany) using a 20× dry objective (HC PL FLUOTAR 20×/0.50 DRY) was performed. A 488 nm and 552 nm laser line were used to excite SYTO9 and propidium iodide, respectively.

### 2.4. Susceptibility Assessment of C. acnes Biofilms to Radial Shockwaves

To assess *C. acnes* susceptibility to rESWT, microorganisms were cultured on Schaedler agar plates and incubated for 72 h at 37 °C anaerobically [21]. Then, the bacterial inoculum was prepared through suspension of bacterial colonies until a turbidity of 0.5 McFarland (approximately 10^8^ CFU/mL) was achieved. Subsequently, *C. acnes* biofilms were grown following incubation for 72 h at 37 °C in the presence of thioglycolate (ThermoScientific, Waltham, MA, USA). Afterwards, rESWT was applied in a fashion similar to that described above (i.e., 1.6 to 2.5 Bar with a frequency of 8–11 Hz and 95 to 143 impulses per disk). To remove free-swimming bacteria and potentially any detached blasting material, the disks were rinsed mechanically three times utilizing NaCl 0.9%. Then, the microtiter plates were covered with Parafilm to achieve watertightness and the disks were sonicated at a frequency of 35 kHz (Transsonic 570 Elma, Singen, Germany) for five minutes at 37 °C to dislodge bacteria from the surface of the titanium disks [23,24]. Thereafter, vortexing was performed and approximately 100μL from the supernatant was transferred from each cell to a new 96-well plate. At the end of this task, the XTT assay was executed and absorbance was read spectrophotometrically at 450 nm.

### 2.5. Treatment Arms

One hundred and twenty disks were sequentially allocated into the following intervention groups: (i) Group A (rESWT at 1.6 Bar with a frequency of 8 Hz and 95 impulses per disk); (ii) Group B (rESWT at 1.6 Bar with a frequency of 11 Hz and 95 impulses per disk); (iii) Group C (rESWT at 2.5 Bar with a frequency of 8 Hz and 95 impulses per disk); (iv) Group D (rESWT at 2.5 Bar with a frequency of 11 hz and 95 impulses per disk); (v) Group E (rESWT at 2.5 Bar with a frequency of 11 Hz, and 143 impulses per disk). For every experiment, we considered *n* = 8 positive (i.e., untreated) and *n* = 8 negative (that is, microorganism-free) control groups to allow for reliable estimation of the antimicrobial efficacy in the intervention groups. Control disks were handled exactly the same with those in treatment groups and one sample was considered in all cases.

### 2.6. Sample Size Calculation and Statistical Analysis

The sample size was calculated in accordance with published guidelines for in vitro trials [25], after accounting for the 80–100% [26,27,28] success rates reported in two-stage revision operations for implant-associated infections. More specifically, a total of at least 20 disks per comparison was calculated with the statistical power set at 0.8 and *α-* and *β- errors* at 5% and 20%, in that order. Statistical analyses were performed using SPSS 25.0 software (SPSS Chicago, IL, USA) and a *p* value of < 0.05 denoted statistical significance. At the beginning of the analyses, a normality test was executed in all cases. If normal distribution was revealed, both parametric and nonparametric tests were considered. For the former, independent-sample t-tests were implemented to compare mean values with their standard errors between intervention and untreated control groups. For the latter, medians were compared between two and multiple groups in terms of the Mann–Whitney and Kruskal–Wallis tests, correspondingly. When data followed non-normal distribution, nonparametric tests were employed, and results were reported by means of medians with the corresponding interquartile ranges. In addition, to achieve comparability in terms of the between-group analysis, we adjusted the spectrophotometric measurements with respect to background correction. To create graphical illustrations, Prism 6 software (GraphPad Software, Inc., La Jolla, CA) was used.

## 3. Results

### 3.1. Antimicrobial Susceptibility of C. acnes Planktonic Form to Radial Shockwaves

**Figure 2:**
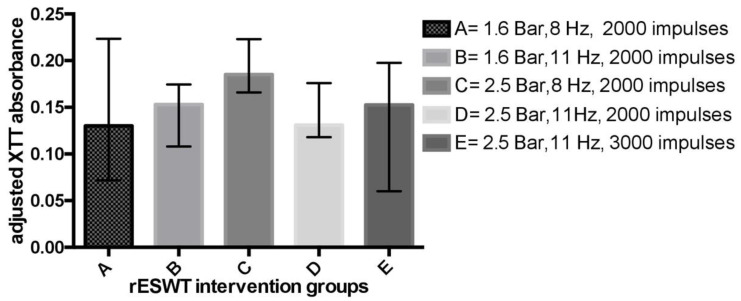
Antimicrobial effects of radial shock waves against *C. acnes* planktonic form expressed as adjusted median absorbances. rESWT = radial extracorporeal shock wave therapy; XTT = 2,3-bis(2-methoxy-4-nitro-5-sulfophenyl)-2H-tetrazolium-5-carboxanilide.

For within-group comparisons, statistically significant differences in favor of intervention groups were detected in all cases (Table 1). By contrast, when multiple-group comparisons were performed, we did not observe any considerable difference among the included treatment arms (adjusted median absorbance (MA) for group A, B, C, D, and E was 0.13 (IQR = 0.0718 to 0.2235), 0.153 (IQR = 0.108 to 0.1745), 0.185 (IQR = 0.166 to 0.223), 0.131 (IQR = 0.118 to 0.176), 0.1525 (0.06 to 0.1975), in that order; *p* = 0.06) (Figure 2). Following the application of rESWT, the reduction in bacterial load compared to untreated control varied from 35% to 54% (Table 1).

### 3.2. Antibiofilm Activity of rESWT

For the first intervention group, statistics showed that there was a considerable difference when compared to untreated control (median absorbance (MA) for the treatment vs control groups were 0.244 (IQR = 0.215–0.282) and 0.298 (IQR = 0.247–0.307), respectively, *p* = 0.033). For the remainder of the intervention arms, we did not detect any significant difference in favor of the treatment groups (*p* > 0.05) (Table 2). The documented reduction in the amount of biofilm, in comparison to untreated control, ranged from 14% to 49% (Table 2).

**Figure 3:**
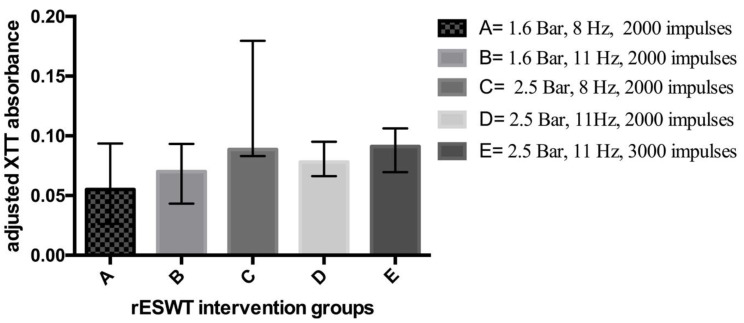
Effects of radial shock waves on *C. acnes* biofilms as measured by adjusted median absorbance. rESWT = radial extracorporeal shock wave therapy; XTT = 2,3-bis(2-methoxy-4-nitro-5-sulfophenyl)-2H-tetrazolium-5-carboxanilide.

For between-group comparisons, a statistically significant difference in favor of a low-energy and low-frequency intervention was demonstrated (adjusted MAs for groups A, B, C, D, and E were 0.055 (IQR = 0.0263 to 0.0935), 0.07 (IQR = 0.0433 to 0.0933), 0.0885 (IQR = 0.083 to 0.1795), 0.078 (IQR = 0.0663 to 0.095), 0.091 (IQR = 0.0695 to 0.1063)), respectively, *p* = 0.007) (Figure 3).

## 4. Discussion

Confronting indolent non-suppurative shoulder PJIs while retaining implants is a challenging task in orthopedic surgery. Owing to the increased revision burden [29] and commonly reported adverse effects stemming from long-term antibiotic suppressive therapy (such as drug resistance, allergic reactions, and toxicity [30,31,32,33]), the adoption of adjuvant modalities, particularly in terms of new nonantibiotic complementary strategies, seems very important [10]. Hence, we investigated the antimicrobial effects of radial shock waves because this is an affordable, relatively new, and not widely investigated intervention [18]. For a realistic and clinically relevant treatment approach of shoulder pathology, we tested the number of impulses as well as frequency and pressure frames recommended by the manufacturer. We demonstrated that up to a 50% reduction in biofilm load was achieved, meaning that, although rESWT cannot be recommended as a standalone treatment, it could be investigated as an adjuvant treatment to antibiotics. Furthermore, in explaining why this method was more successful with freely moving bacteria rather than microorganisms enclosed within a polymer matrix, we advocate that the biofilm structure provided protection to *C. acnes* similar to that occurring following antibiotic administration [34].

### 4.1. Biomaterial and Strain Selection

In the current in vitro study, we used sandblasted titanium disks because titanium and its alloys have been extensively used in shoulder-related surgery in recent decades [35]. In particular, this biomaterial is commonly considered in cementless shoulder arthroplasty as well as fracture fixation of proximal humeral fractures, with a proven track record of chemical stability and biocompatibility [36,37]. Concerning the roughness of the employed titanium disk surfaces, we considered standard roughened biomaterials [38] (i.e., disks with poor light reflective capacity) that consistently present higher bacterial adhesion when compared to smoother surfaces [36,39,40,41]. Given the fact that there is no consensus in the literature on an optimum titanium surface roughness [42], we aimed to achieve a S_a_ of more than 2 μm [43]. It is also important to note that, in the presence of a titanium implant, a surface protein layer is physiologically formed which subsequently renders the surface of the biomaterial prone to biofilm development [38].

As per our confocal laser microscopy biofilm evaluation, standard *C. acnes* strain was capable of exhibiting substantial contamination potential featuring a massive living cell population. This was because biofilm formation is undoubtedly a distinctive feature of invasive subtypes. Those subtypes are commonly isolated from device-associated infections, as opposed to skin isolates [44]. Besides, when it comes to studying biofilms produced by clinical isolates, recent evidence has suggested that variant distributions and proportions of living and dead *C. acnes* cells are more often than not detected [45].

### 4.2. rESWT Application and Reliability in Cell Viability Measurements

When it comes to shock wave treatment application in clinical practice, an ultrasound gel over the area under treatment is usually utilized to achieve sufficient energy conduction. Similarly, in the laboratory setting, we applied an ultrasound gel to the top of 96-well plates and pointed the ultrasound head towards the titanium disks vertically to ensure even energy distribution.

Concerning credibility of the reported findings, evidence has suggested that the XTT ([2–bis(2-methoxy-4-nitro-5-sulfophenyl)-2H-tetrazolium-5-carboxanilide]) reduction assay is a highly reliable method as compared to CFU counting [22,46,47] and particularly suitable for implementing multiple technical replications (i.e., repetition of the same condition within the same experiment).

### 4.3. Effect of rESWT against C. acnes Planktonic and Biofilm Forms

Regarding the impact of radial shock waves on free-swimming *C. acnes*, bacteria, there was a lack of “dose-response” effect in all cases. Statistically significant differences relative to untreated control were demonstrated regardless of the variability in the frequency, number of impulses, and pressure amongst the treatment groups. By contrast, concerning the impact of rESWT on *C. acnes* biofilms, only a low-pressure and low-frequency setting with a low number of impulses yielded a statistically significant difference. In particular, a 50% reduction in the amount of biofilm was documented compared to untreated control, when a 1.6-Bar setting with a frequency of 8 Hz and 2000 impulses per implant was used. However, for the treatment outcome to be clinically relevant, total elimination of all viable bacteria is required because, otherwise, infection can propagate [48]. Based on the above, rESWT cannot be recommended as a standalone treatment against *C. acnes* implant-related infections. Nevertheless, it may be considered as an adjuvant modality to antibiotics. We also demonstrated that it is not necessary to raise the shock wave pressure, frequency, and number of impulses, with the aim to further enhance its anti-bacterial efficacy, thus minimizing the risk of associated post-intervention adverse events such as regional pain, soft tissue hematoma, petechia, and microfractures, in case of clinical use in the future.

### 4.4. Study Limitations and Implications for Future Research

While data generated by in vitro models of infections provide useful insights into *C. acnes* biofilm behavior, we should be careful when extrapolating the results from the current study to deep infections in humans with implants featuring complex geometry. To elaborate, when it comes to assessing device-associated infections, multiple clinical factors should be borne in mind, including, but not limited to, bacterial load, implant characteristics, host immune system status, nutrition, pH, strain virulence, and specific adhesion molecules [15]. Therefore, to account for the above clinical complexity and determine whether the mean radial shock wave penetration depth of 3 cm is sufficient to reach all parts of implants embedded in bone, we suggest that rabbit models of infection be utilized at a subsequent research stage [49].

Furthermore, while it has been claimed that localized infections should no longer be considered a contraindication to shock wave application in orthopedics [50], large-scale studies with long-term follow-up observations should be conducted before drawing safe conclusions on ESWT’s track record of safety [51]. A further potential complication which is worth investigating is contribution of shock waves to implant aseptic loosening.

Additionally, consideration of multiple rESWT sessions to optimize its antimicrobial effects could possibly be considered. For instance, application of rESWT at weekly intervals may be employed as per manufacturer’s recommendations with the aim to examine whether or not the effects of this treatment are cumulative.

Furthermore, it should be noted that following shock wave application, some remaining sandblasting material is likely to detach from titanium disks, thus making suspension cloudier and potentially interfering with cell viability measurements. This potential turbidity interference could have affected the results of planktonic susceptibility testing as the supernatant was utilized to assess the shock wave treatment effect relative to untreated control. By contrast, in biofilms experiments, this issue was overcome by meticulous mechanical rinsing of the disks prior to sonication.

Finally, according to our findings, we suggest that combinations of biofilm-validated antibiotics with shock wave therapy should be further investigated against *C. acnes* biofilms, as recent in vitro studies have suggested that such treatment combinations are highly effective against biofilms produced by clinically relevant aerobic bacteria [12,52,53].

## 5. Conclusions

According to our findings, low-pressure/low-frequency rESWT caused a 50% reduction in the formation of biofilms of *C. acnes* in vitro. Therefore, rESWT could be further investigated in vivo, as an adjuvant treatment to antibiotics, as it may result in higher eradication rates against *C. acnes* implant-associated infections.

## Figures and Tables

**Table 1:** Antimicrobial effects of radial Extracorporeal Shock Wave Treatment on *C. acnes* planktonic form.

Treatment Group (*n*)	Mean Absorbance Difference * (SE)	*p* Value for Parametric Tests ^†^	Median Absorbance (IQR)		*p* Value for Non-Parametric Tests ^†^	% Reduction in Bacterial Load Based on Negative Control Measurements (Median)
			Intervention Group	Positive Control Group		
A (20) (8 Hz, 1.6 Bar, 2000 impulses)	0.1995 (0.044)	0.000175	0.7705 (0.712 to 0.864)	0.9245 (0.888 to 0.104)	0.001	54% (0.64)
B (20) (11 Hz, 1.6 Bar, 2000 impulses)	N/A	N/A	0.7937 (0.748 to 0.815)	0.9245 (0.888 to 0.104)	0.001	46% (0.64)
C (20) (8 Hz, 2.5 Bar, 2000 impulses)	0.138 (0.346)	0.001	0.8255 (0.807 to 0.863)	0.9245 (0.888 to 0.104)	0.001	35% (0.64)
D (20) (11 Hz, 2.5 Bar, 2000 impulses)	0.188 (0.037)	*p* = 0.000044	0.7720 (0.758 to 0.817)	0.9245 (0.888 to 0.104)	0.0003	46% (0.64)
E (20) (11 Hz, 2.5 Bar, 3000 impulses)	0.255 (0.055)	*p* = 0.000121	0.8870 (0.795 to 0.932)	1.0900 (1.014 to 1.240)	0.001	42% (0.61)

Statistically significant differences between intervention and control groups are demonstrated, suggesting antimicrobial efficacy of rESWT against *C. acnes* free-floating cells; IQR = interquartile range; rESWT = radial extracorporeal shock wave therapy; N/A = Not applicable (due to data following non-normal distribution); SE = Standard Error between intervention and untreated control groups; ^†^ *p* < 0.05 indicates significance. * between intervention and untreated control groups.

**Table 2:** Antibiofilm activity of rESWT on *C. acnes* biofilms.

Treatment Group (*n*)	Mean Absorbance Difference (SD)	*p* Value for Parametric Test ^†^	Median Absorbance (IQR)		*p* Value for Nonparametric Test ^†^	% Reduction in Biofilm Based on Negative Control Measurements (Median)
			Intervention Group	Untreated Control Group		
**A (24)** **(8 Hz, 1.6 Bar, 2000 impulses)**	**N/A**	**N/A**	**0.244 (0.215–0.282)**	0.298 (0.247–0.307)	**0.033**	49% (0.188)
B (24) (11 Hz, 1.6 Bar, 2000 impulses)	0.0005 (0.084)	0.973	0.255 (0.228–0.278)	0.266 (0.246–0.277)	0.571	14% (0.187)
C (24) (8 Hz, 2.5 Bar, 2000 impulses)	N/A	N/A	0.280 (0.275–0.371)	0.308 (0.264–0.369)	0.421	25% (0.196)
D (24) (11 Hz, 2.5 Bar, 2000 impulses)	N/A	N/A	0.266 (0.254–0.283)	0.297 (0.252–0.33)	0.306	29% (0.19)
E (24) (11 Hz, 2.5 Bar, 3000 impulses)	N/A	N/A	0.331 (0.31–0.346)	0.365 (0.340–0.377)	0.112	24% (0.223)

Results on statistical comparisons between intervention and untreated control groups are shown; a statistically significant difference in favor of comparison 1 is displayed in bold; IQR = interquartile range; N/A = Not Applicable (due to data following non-normal distribution); SD = Standard Deviation; rESWT = radial extracorporeal shock wave therapy; ^†^ *p* < 0.05 denotes statistical significance.

## Supplementary Materials

The following are available online at https://www.mdpi.com/2076-2607/8/5/743/s1, Figure S1: A sandblasted titanium alloy (Ti6Al4V) disk (diameter 4 mm, height 2 mm) is depicted.
