# Supplementary material for: Radial Extracorporeal Shock Wave Therapy against Cutibacterium acnes Implant-Associated Infections: An in Vitro Trial
Source: Microorganisms. 2020 May 15;8(5):743. doi: 10.3390/microorganisms8050743 (PMC7285346; doi:10.3390/microorganisms8050743)
Supplement: Supplementary file 1 [file microorganisms-08-00743-s001.pdf]

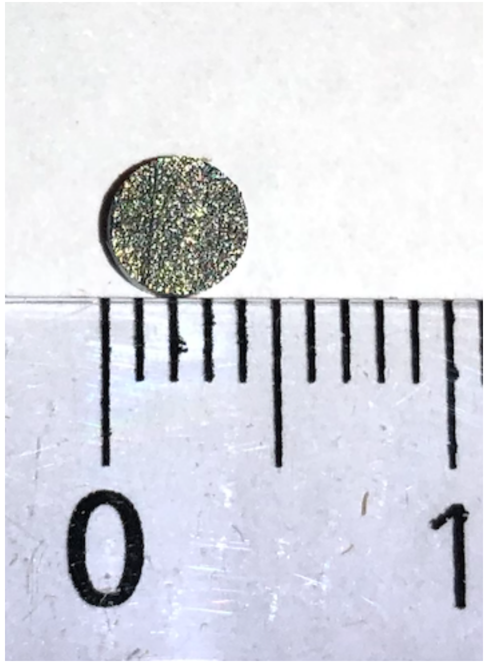

Supplemental Figure 1. A sandblasted titanium alloy (Ti6Al4V) disk (diameter 4mm, height 2mm) is depicted
